# Lytic Susceptibility, Structure, and Mechanical Properties of Fibrin in Systemic Lupus Erythematosus

**DOI:** 10.3389/fimmu.2019.01626

**Published:** 2019-07-16

**Authors:** Rustem I. Litvinov, Rosa M. Nabiullina, Laily D. Zubairova, Mileusha A. Shakurova, Izabella A. Andrianova, John W. Weisel

**Affiliations:** ^1^Department of Cell and Developmental Biology, University of Pennsylvania Perelman School of Medicine, Philadelphia, PA, United States; ^2^Institute of Fundamental Medicine and Biology, Kazan Federal University, Kazan, Russia; ^3^Departments of Biochemistry and General Pathology, Kazan State Medical University, Kazan, Russia

**Keywords:** fibrinogen, fibrin structure, fibrin viscoelasticity, fibrinolysis, systemic lupus erythematosus

## Abstract

Among complications of systemic lupus erythematosus (SLE), thrombotic events are relatively common and contribute significantly to the morbidity and mortality rates. An increased risk of thrombosis in various diseases has been shown to be associated with the lytic stability and mechanical stiffness of the fibrin clot determined by its structure. Here we studied alterations of the fibrin clot properties in relation to disease severity in SLE patients. Plasma clots from 28 SLE patients were characterized by the kinetics of formation and fibrinolytic dissolution (using dynamic turbidimetry), the network and fiber ultrastructure (scanning electron microscopy), viscoelasticity (shear rheometry), and the rate and degree of crosslinking (Western blotting) correlated with the disease activity, blood composition, and compared to clotting of pooled normal human plasma. Clots made from plasma of SLE patients were lysed faster with exogenous t-PA than control clots from normal plasma without a significant difference between those from active (SLEDAI>4) and inactive (SLEDAI<4) SLE patients. Clots from the blood of patients with active SLE were characterized by significantly slower onset, but faster rate of fibrin polymerization and a higher optical density due to thicker fibers compared to those from inactive SLE and control pooled normal plasma. The rheological parameters of the clots (storage and loss moduli) were significantly increased in the active SLE patients along with enhanced fibrin crosslinking and hyperfibrinogenemia. The structural and rheological alterations displayed a strong positive correlation with high fibrinogen levels and other laboratory markers of immune inflammation. In conclusion, changes in the blood composition associated with active systemic inflammation in SLE cause significant alterations in the lytic resistance of fibrin clots associated with changes in polymerization kinetics, viscoelastic properties, and structure. The formation of more rigid prothrombotic fibrin clots in the plasma of SLE patients is likely due to the inflammatory hyperfibrinogenemia and greater extent of crosslinking. However, the higher susceptibility of the SLE clots to fibrinolysis may be a protective and/or compensatory mechanism that reduces the risk of thrombotic complications and improves patient outcomes.

## Introduction

Systemic lupus erythematosus (SLE) is a chronic autoimmune disease with possible involvement of various organs and systems as well as varying manifestations and severity of the clinical course and outcomes. The prevalence of SLE is about 20–150 per 100,000 (1, 2), it mainly affects people between 15 and 44 years of age, and the 10-year survival rate varies between 70 and 90% (3, 4). Despite the improvements in care, patients often suffer long-term morbidity that adversely affects their quality of life, resulting in substantial direct and indirect costs (4). Clinical presentations of SLE are highly diverse and include mucocutaneous, musculoskeletal, renal involvement as well as neuropsychiatric, pulmonary, gastrointestinal, and cardiac abnormalities.

Thrombotic events are quite common in SLE, with the incidence that is 25- to 50-fold higher than in the general population (5) indicating that SLE itself is an independent risk factor for developing both arterial and venous thrombosis. The incidence of thrombosis depends on the clinical and laboratory characteristics of SLE and could further increase when SLE is associated with other general, demographic risk factors or with inherited or acquired pro-thrombotic abnormalities (6–8). Thrombosis appears to be one of the most common causes of death in SLE; thrombotic events and cardiovascular accidents can be the first complications of SLE after reactivation of the disease (3, 9).

Mechanisms underlying thrombosis in SLE are complex and still not clear. Most commonly, thrombosis is associated with antiphospholipid antibodies (aPL Abs) directed against phospholipids and protein antigens, including pro- and anticoagulants (10). However, it was recently shown that clinically significant aPL Abs are present only in the blood of one third of SLE patients (11); therefore, mechanisms of thrombosis other than those mediated by aPL Abs should be also considered. Systemic endothelial damage and inflammation induced by circulating immune complexes are strongly associated with hypercoagulability revealed in SLE by multiple molecular markers, such as elevated protein C activity, high levels of antithrombin, thrombin-antithrombin complexes, and D-dimer (12–14). Impaired fibrinolysis is another pathogenic mechanism in SLE, which may help explain why these patients are at increased risk of developing thrombosis (15). Defects in fibrinolysis are related to an increase in the levels of various fibrinolytic inhibitors, such as PAI-1 (16), α2-antiplasmain, TAFI (17), lipoprotein (a) (18), and a decrease of plasminogen activity (17) likely due to anti-plasminogen antibodies (19).

An important player in hemostasis and thrombosis is fibrin, a major component and the mechanical scaffold of blood clots and thrombi (20, 21). Proteolytic action of thrombin initiates fibrinogen conversion to fibrin and formation of a space-filling branched polymeric fibrin network (20). The meshwork of fibrin fibers forms the primary structural basis of a hemostatic blood clot or an obstructive thrombus. Fibrin fibers can differ in thickness, length, number of branch points, which determine the network density, its mechanical properties and susceptibility to enzymatic lysis. Various genetic and environmental factors may alter clot structure and stability, such as fibrinogen and thrombin concentrations, inflammation-associated reactive oxygen species, nitrating metabolites, antibodies, neutrophil extracellular traps, etc (20, 22–25). Changes of fibrin clot microstructure that determines susceptibility to fibrinolysis and mechanical properties underlie the course and outcomes of hemostatic disorders, including thromboembolism (26, 27). Abnormal final network structure and increased stability of fibrin clots is associated with thrombosis (28). Patients with thrombotic disorders form plasma clots *in vitro* with an altered “prothrombotic” phenotype, meaning that these clots are more rigid, dense, less permeable and lyse slower than those formed in the blood of healthy subjects (29). Thus, characterization of clot structure is of great clinical significance since it correlates with epidemiological and clinical data in various diseases.

Among systemic immune-related connective tissue diseases, only rheumatoid arthritis, antiphospholipid syndrome, and eosinophilic granulomatosis with polyangiitis were shown to be associated with decreased fibrin clot permeability and lysability (30–32), while only an altered fibrin structure was shown in clots from SLE patients (33). Because thrombosis is a frequent and life-threatening complication in SLE, investigation of the fibrin clot properties in SLE patients is important both pathophysiologically and clinically as it may help to understand the pathogenesis and develop new strategies of prophylaxes and treatments. Here we studied alterations of fibrin clot formation and properties in the blood of SLE patients in relation to the disease severity.

## Materials and Methods

### Patients

Twenty eight consecutive patients, female/male 82/18 (%) at a mean age of 37 ± 13 years, were included in the study based on the revised criteria of the American College of Rheumatology for SLE (34–36). Informed consent was obtained from the SLE patients and healthy donors under approval of the Ethical Committee of the Kazan State Medical University. All procedures were carried out in accordance with the approved guidelines. The disease activity was assessed using the SLE disease activity index (SLEDAI-2K) (35, 36). Based on the disease activity score, the patients were categorized into two groups: 14 (50%) patients with SLEDAI<4 comprised the “inactive” group and 14 (50%) patients with SLEDAI>4 formed the “active” group. The main clinical and laboratory characteristics of the patients are summarized in Tables 1, 2. SLE patients were excluded from this study if they had any acute comorbidity, cancer, hepatic injury, a chronic kidney disease stage 4 or more, or if they were given anticoagulants, thrombolytics or antiplatelet drugs within 2 weeks prior to examination. All the patients were on corticosteroids and two of them were on Plaquenil. Antiphospolipid syndrome was diagnosed in 3 women based on a history of deep vein thrombosis (2 patients) and recurrent miscarriage combined with positive blood tests for lupus anticoagulant (2) and anticardiolipin antibodies (37).

**Table 1:** Clinical characteristics of the SLE patients at the time of examination.

**Clinical manifestations**	**Active SLE - SLEDAI>4**	**Inactive SLE - SLEDAI <4**
	**(*n* = 14)**	**(*n* = 14)**
Anti-phospholipid syndrome	18%	0
Lupus nephritis	54%	36%
Chronic renal failure	45%	9%
Musculoskeletal features (arthritis, arthralgia, osteoarthrosis, osteoporosis, myopathy)	73%	36%
Mucocutaneous features (erythema, photodermatosis, hemorrhagic purpura, Raynaud phenomenon)	45%	36%
Cardiovascular features (pericarditis, vasculitis, arterial hypertension, valvular heart disease)	54%	27%
Neurological manifestations (status epilepticus, migraines, cervicogenic headache, cognitive disorders)	36%	9%
Pulmonary manifestations (acute bronchitis, pleuritic chest pain, chronic obstructive pulmonary disease, respiratory insufficiency)	27%	0
History of thrombosis (8 months and 2 years ago)	14%	0

*The total percentage is >100% because some patients had more than one clinical manifestation*.

**Table 2:** Laboratory parameters (mean ± SD) in patients with SLE (*n* = 28).

**Parameters (in parentheses - reference values)**	**Patients with SLE**
**Hemostatic Parameters**	
APTT (28–40), s	22 ± 6.9
Fibrinogen (2–4), g/L	3.1 ± 1.2
Prothrombin time (9.2–12.2), s.	13.7 ± 2.5
INR (0.9–1.5)	1 ± 0.1
**Hematologic Parameters**	
Platelet count (180–300), × 10^9^/L	268 ± 70
Red blood cells (3.5–5.5), × 10^12^/L	4.5 ± 0.6
Leukocytes (4–10), × 10^9^/L	8.1 ± 4.7
Eosinophils (0.5–5), %	1.9 ± 1.8
Monocytes (3–11), %	7.2 ± 3.9
Lymphocytes (19–37), %	28 ± 10
Basophils (0–1), %	0.4 ± 0.7
Neutrophils (47–87), %	64 ± 10
Hemoglobin (115–160), g/L	132 ± 14
ESR (2–20), mm/h	24 ± 8
**Biochemical Parameters**	
Total protein (67–87), g/L	66 ± 10
Albumin (35–52), g/L	45 ± 15
Glucose (3.5–5.9), mmol/L	4.8 ± 1.2
Cholesterol (3–5.2), mmol/L	5.2 ± 1.4
ALT (<45), U/L	12.6 ± 3.7
AST (<35), U/L	15.4 ± 3.8
Total bilirubin (<21), μmol/L	6.6 ± 3.2
Urea (3.5–8.3), mmol/L	5.6 ± 3.6
Creatinine (60–120), μmol/L	73 ± 36
Uric acid (200–430), μmol/L	479 ± 126
K^+^ (3.4–5.1), mmol/L	4.2 ± 0.9
Na^+^ (136–146), mmol/L	143 ± 3
Ca^2+^ (2.3–2.8), mmol/L	1.6 ± 0.6
**Immunological Parameters**	
Anti-β2-glycoprotein Abs (0–20), U/ml	12.2 ± 9.4
Anti-dsDNA Abs (<25), U/ml	97.1 ± 88.2
IgA (1.1–3.5), mg/mL	2.2 ± 1.5
IgM (0.7–2.5), mg/mL	1.6 ± 0.7
IgG (6.7–16.5), mg/mL	13.8 ± 6.3
CICs (<50), mg/L	103 ± 72
Total complement activity (31–60), U/mL	23.8 ± 10.0

### Blood Collection and Processing

Blood was drawn under aseptic conditions without venous stasis using vacutainers containing 3.8% trisodium citrate (Greiner Bio-One, Austria) at 9:1 v/v. For investigations of plasma clots, the blood was centrifuged at room temperature for 15 min at 1,500 g to obtain platelet-poor plasma, then 5 min at 10,000 g to obtain platelet-free plasma, which was then aliquoted and stored at −80°C until use. For control experiments, platelet-free plasma from at least 10 healthy donors was pooled, aliquoted and stored at −80°C until use. The frozen samples were thawed at 37°C for 60 min and used within 2 h. A part of each individual blood sample was used for standard immunological, hematological, and biochemical tests.

### Turbidimetric Fibrinolysis Assay

**Figure 1:**
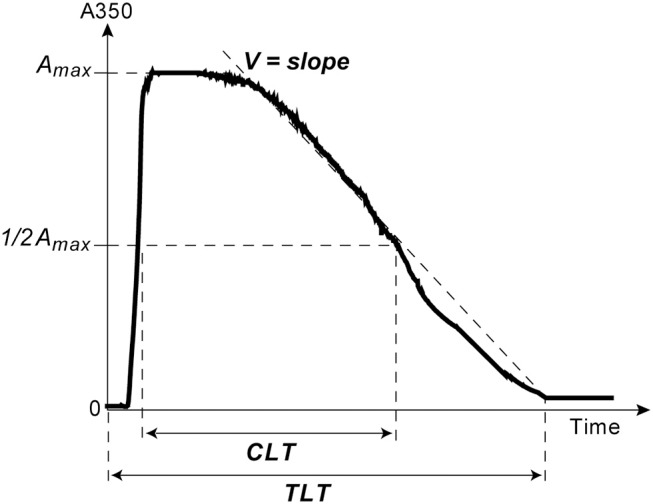
A typical turbidimetry curve of fibrin formation and lysis with the following extracted parameters of intrinsic fibrinolysis: (1) Total Lysis Time (*TLT*), i.e., the time from adding CaCl_2_ and t-PA until the time point when the OD almost reaches the baseline and does not change any more, which corresponds to complete clot dissolution; (2) the rate of fibrin lysis (*V*), i.e., the slope of the descending limb of the curve between the end of the horizontal plateau and the end point of TLT; (3) the time needed to reduce the maximum turbidity of the clot to the half-maximal value (t1/2); (4) Clot Lysis Time (*CLT*), which was defined as the time from the midpoint of the clear to maximum turbid transition to the midpoint in the transition from maximum turbidity to the final baseline turbidity (38).

The kinetics of clot lysis was studied in general accordance with the recommendations of the Scientific and Standardization Committee of the International Society on Thrombosis and Haemostasis on the clot turbidity and lysis assay, but without using thrombin (38). Plasma clotting and fibrinolysis were initiated by the simultaneous addition of 100× Milli-Q water solution of CaCl_2_ (24 mmol/l final concentration) and t-PA (HYPHEN BioMed, France) (50 ng/ml final concentration) to induce lysis of the clot from inside throughout the entire volume. Fibrin formation followed by dissolution was monitored at 37°C by a Shimadzu UV-1800 (Japan) spectrophotometer at λ = 350 nm in a thermostatic cuvette. From the turbidimetric lysis curves, the following variables were determined (Figure 1): (i) total lysis time (*TLT*), i.e., the time needed for complete clot dissolution; (ii) the 50% clot lysis time (*CLT*) or the time from the midpoint of the clear to maximum turbid transition to the midpoint in the transition from maximum turbidity to the final baseline turbidity; (iii) the rate of fibrin lysis (*V*), i.e., the decrease of the optical density per unit of time in the linear descending part of the curve, just after the plateau.

### Turbidimetric Clotting Assay

**Figure 2:**
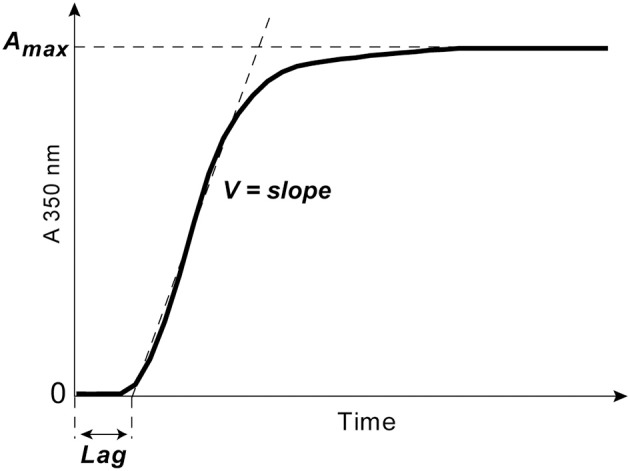
A typical turbidimetry curve with the following extracted parameters of fibrin formation: (1) the lag phase (*Lag*) or the time from adding CaCl_2_ until an increase of the optical density by 0.01, which measures the time needed for thrombin generation and protofibril formation; (2) the slope of the curve or the rate of polymerization (*V*) taken between the end of the lag phase through the linear part of the curve, which measures the velocity of lateral aggregation of protofibrils and fiber formation; (3) the maximum optical density (A_*max*_) at the plateau, which reflects the amount of fibrin formed and the fibrin fiber cross-sectional area.

Clotting of citrated platelet-free plasma induced by adding 100× water solution of CaCl_2_ at a 24 mmol/l final concentration without adding thrombin was followed by monitoring continuously the optical density at λ = 350 nm at 37°C using a Shimadzu UV-1800 (Japan) spectrophotometer. From the turbidimetry curves, the following parameters were determined: (i) the lag time (*Lag*) before turbidity starts to increase after initiation of clotting; it measures the time needed for thrombin generation and protofibril formation; (ii) the slope of the curve or the rate of polymerization (*V*), which measures the velocity of lateral aggregation of protofibrils and fiber formation; (iii) the maximum optical density (A_*max*_) at the plateau, which reflects the amount of polymerized protein and fibrin network structure particularly fiber thickness and network density (Figure 2).

### Scanning Electron Microscopy of Fibrin Clots

Fibrin clots formed by adding CaCl_2_ (24 mmol/l final concentration) without thrombin to platelet-free plasma were allowed to form for 2 h at room temperature in a humid chamber. The clots were washed in 0.05 M sodium cacodylate buffer (pH 7.4), then fixed in 2% glutaraldehyde in the same buffer, dehydrated in ethanol, dried with hexamethyldisilazane, and sputter coated with gold-palladium. The samples were examined in a Merlin (Carl Zeiss, Germany) scanning electron microscope. Fiber thickness was measured using the Adobe Photoshop CS4 software in randomly selected areas for a total of 100 fibers per clot (3 clots per each experimental condition).

### Shear Rheometry of Fibrin Clots

Viscoelastic properties of fibrin clots were studied in a DHR-2 rheometer (TA Instruments, New Castle, USA) with a horizontal plate-plate geometry (plate diameter 25 mm). One hundred fifty microliter of platelet-free plasma was activated by adding CaCl_2_ (24 mmol/l final concentration) and phospholipids (cephalin at 1 μg/μl final concentration) and placed to form a clot in the gap of the rheometer at 37°C. The addition of cephalin standardized and accelerated the time of clot formation to prevent it from drying before the measurements started. The edge of the gap was sealed with mineral oil to prevent evaporation and the samples were allowed to polymerize for 30 min. Measurements were taken in a shear mode using a strain of 3% and frequency 5 rad/s for 1 min, the time sufficient to get the measured parameters stable. Using the software TRIOS V2.0 (TA Instruments), the following viscoelastic parameters were extracted: the storage modulus, *G*′, reflecting reversible mechanical deformation or stiffness, and the loss modulus, *G*″, characterizing irreversible deformation or plasticity. The loss tangent (*tan* δ = *G*″*/G*′*)* was calculated as a measure of the energy loss against energy stored during deformation.

### Western Blot Analysis of the Fibrin Crosslinking Kinetics

To see if the kinetics of factor XIIIa-catalyzed fibrin γ-γ-crosslinking is changed in SLE compared to normal clots irrespective of the endogenous thrombin activity, platelet-free plasma samples in 9 μl aliquots were clotted by adding 1 μl CaCl_2_ (25 mM final) and human thrombin (0.1 U/ml final). The crosslinking reactions in each sample were quenched at the time points of 1, 5, 15, 30, 45, 60, 90, and 120 min with an equal volume of 8 M urea, 1% SDS, and 1% dithiothreitol followed by boiling at 100°C for 5 min. Samples were run on a 9% SDS-PAGE followed by transfer of proteins to polyvinylidene difluoride membrane (EMD Millipore) and probing with monoclonal mouse anti-human fibrinogen antibody (1:8000) (Abcam) and secondary goat anti-mouse IgG antibodies horseradish peroxidase conjugate (1:2500) (Invitrogen). The blots were viewed and quantified by the ChemiDoc™ XRS+ system (Bio-Rad). γ-Dimer formation was normalized for the sum of γ-chain and γ-dimer bands at each time point, respectively. With the antibodies used in this assay, Western blot signals from the bands of both uncrosslinked α-chains and crosslinked α-chain polymers were weak and not quantifiable.

### Statistical Analysis

For parametric distributions comparing two groups, a Student's *t*-test was performed. For comparisons between more than two groups, one-way ANOVA was used for parametric data, followed by Tukey's multiple-comparison tests. Values are expressed as a mean ± standard deviation. Correlations were assessed by Spearman rank correlation analysis. *P*-values are reported for comparison between groups as follows: ^*^p < 0.05, ^**^p < 0.01, and ^***^p < 0.001. All statistical calculations were generated and analyzed using the Microsoft Excel (Microsoft) and GraphPad Prism software.

## Results

### Reduced Lytic Stability of Fibrin in SLE

**Figure 3:**
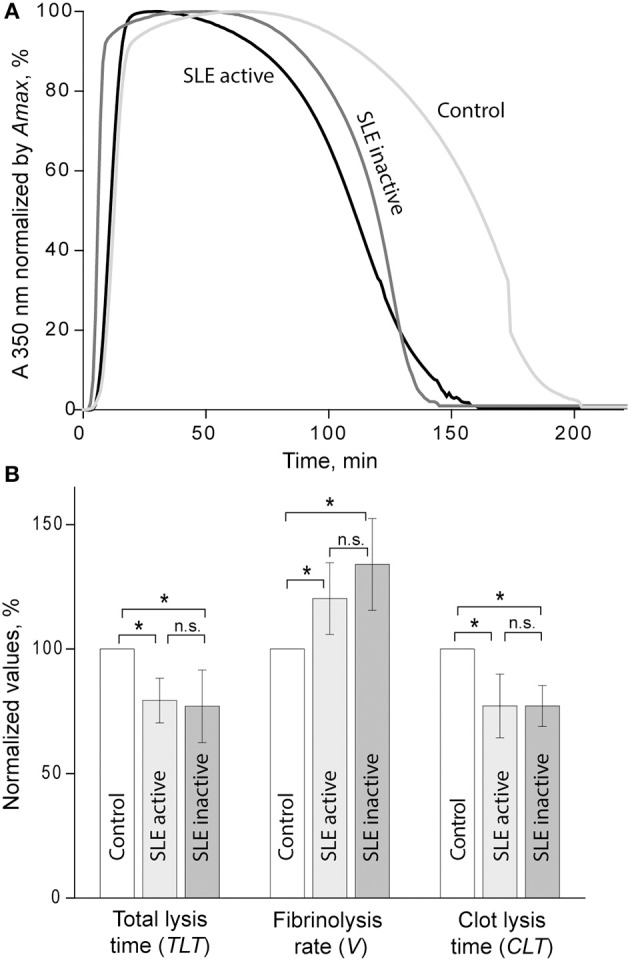
**(A)** Averaged curves of clot formation and lysis in control and SLE plasma samples from patients with inactive (SLEDAI<4) and active (SLEDAI>4) SLE. Clots were lysed by adding equal amounts of t-PA to the plasma samples before clotting was initiated by recalcification. **(B)** Comparative parameters of fibrinolysis (for definitions see Figure 1). The results are presented as a normalized mean value ± SD from the measurements performed in control pooled normal plasma (taken as 100%) compared to the inactive (*n* = 9) and active (*n* = 9) SLE patients. ^*^p < 0.05.

Susceptibility of clots to enzymatic lysis was studied with the dynamic turbidity of re-calcified plasma to which exogenous t-PA was added before the initiation of clotting, thus mimicking natural pathophysiological dissolution of clots and thrombi in the absence of thrombolytic therapy. The amount of t-PA was adjusted to trigger fibrinolysis only after the absorbance reached a plateau of maximum turbidity to ensure that the polymerization was complete, and that t-PA did not affect clot formation. The rate of lysis was significantly faster in SLE samples compared to samples from healthy controls. In SLE in comparison to control, respectively, the clot lysis time (118 ± 31 min. vs. 141 ± 13 min., *p* < 0.05) and the total lysis time (170 ± 42 min. vs. 202 ± 25 min., *p* < 0.05) were significantly shortened while the average rate of lysis (0.84 ± 0.21 vs. 0.53 ± 0.08, *p* < 0.001) was accelerated. It has been shown previously that the rate of fibrinolysis measured as a slope of the descending part of the optical curve depends on the absolute maximum turbidity, which may be a source of inaccuracy (38). To correct for this potential imprecision, we normalized individual kinetic curves by the maximum turbidity taken as 100% and recalculated the parameters of fibrinolysis, which confirmed the accelerated rate of fibrinolysis in the SLE samples compared to control (Figure 3). Remarkably, there was no significant difference in fibrinolysis characteristics between the clots in active (SLEDAI>4) and inactive (SLEDAI<4) forms of SLE. Taken together, these results indicate that fibrin clots from SLE patients are more susceptible to the t-PA-induced internal fibrinolysis than clots from healthy subjects and that this higher proteolytic susceptibility is maintained irrespective of the degree of disease activity. To establish the relationship between the lytic susceptibility and quantitative and/or qualitative changes in fibrin clots made from the blood of SLE patients, we analyzed the kinetics of fibrin formation, clot structure, the extent of crosslinking, and mechanical properties with respect to variations in fibrinogen concentrations and other clinical and laboratory features of SLE.

### Kinetics of Clot Formation and the Maximum Optical Density in SLE Plasma Samples

The turbidimetric clotting assay revealed dramatic differences in the rate of fibrin formation and optical density of clots formed from plasma of the entire cohort of SLE patients compared to controls. The average lag time, i.e., the time required for thrombin generation and formation of fibrin protofibrils after re-calcification, was appreciably prolonged in SLE patients compared to controls (14.6 ± 9.7 min vs. 3.7 ± 0.4 min, respectively, *p* < 0.05). No significant differences in the polymerization rate in SLE compared to control were found. The maximum optical density of the SLE clots was significantly higher than those of controls (0.71 ± 0.28 vs. 0.50 ± 0.01, respectively, *p* < 0.05). After correction for fibrinogen concentration (A_max_/[Fg]), the difference in the maximum optical density remained statistically significant (0.25 ± 0.05 for SLE and 0.19 ± 0.01 for control, *p* < 0.001), suggesting that it was due to variations in fibrin structure rather than the amount of protein in the clots.

**Figure 4:**
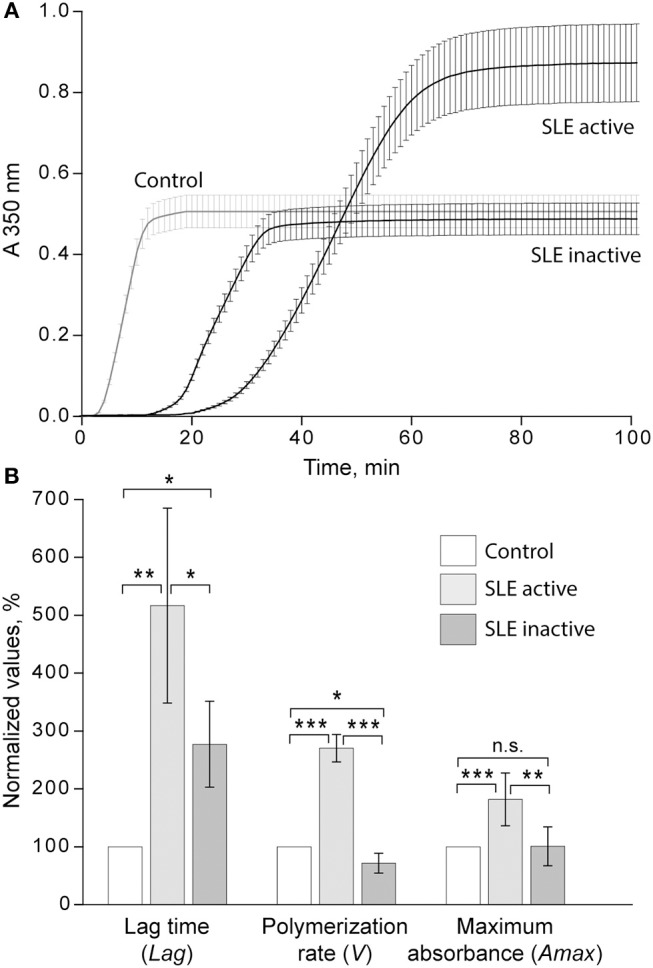
**(A)** Averaged dynamic turbidity curves obtained upon re-calcification of control plasma samples and plasma from patients with inactive (SLEDAI<4) and active (SLEDAI>4) SLE. **(B)** Comparative parameters of the dynamic turbidimetry (for definitions see Figure 2). The results are presented as a normalized mean value ± SD from the measurements performed in control pooled plasma (taken as 100%) parallel to the SLE patients in the active (*n* = 14) and inactive (*n* = 14) forms of the disease. ^*^p < 0.05, ^**^p < 0.01, ^***^p < 0.001.

When the kinetic parameters of clot formation and optical density were normalized by the control and analyzed with respect to the disease activity, there was a striking difference between patients with inactive and active SLE (Figure 4A). In the patients with active SLE (SLEDAI>4), the average lag time was prolonged >2-fold (*p* < 0.01), the polymerization rate was significantly faster (*p* < 0.001), and the maximum optical density was significantly higher (*p* < 0.01) as compared to inactive SLE (SLEDAI<4). When compared to control plasma samples, in the samples from patients with inactive SLE the lag time was moderately but significantly prolonged (*p* < 0.05), the polymerization rate was moderately but significantly slower (p < 0.05), and the final optical density of clots was not different from the control (Figure 4B).

It is noteworthy that the variations of A_max_ revealed in plasma clots followed the same trend as the average concentrations of fibrinogen [Fg]. Specifically, in the active SLE plasma samples the average [Fg] was significantly higher than in controls (3.8 ± 1.2 g/l vs. 2.6 ± 0.05 g/l, respectively, *p* < 0.05), while in the inactive SLE group the fibrinogen level was unchanged compared to controls (2.5 ± 0.7 g/l vs. 2.6 ± 0.05 g/l, respectively, *p* > 0.05). After correction for the fibrinogen concentration (A_max_/[Fg]) in each individual plasma sample analyzed, the average maximum optical density of the SLE clots was still significantly different from controls (0.25 ± 0.05 vs. 0.19 ± 0.01, respectively, *p* < 0.001), implying that it reflects distinctions in fibrin structure rather than in the concentration of fibrin in the clots. Remarkably, the parameters of clot turbidimetry correlated well with the protein composition of blood (Table 3) as well as with the viscoelastic characteristics of the same plasma clots (Table 4). Namely, the maximum optical density displayed direct and strong correlation with the laboratory markers of immune inflammation, such as fibrinogen, erythrocyte sedimentation rate, α-2 globulin, β-1 globulin, β-2 globulin, and circulating immune complexes. Notably, there was a significant inverse correlation of the clot optical density with the level of albumin.

**Table 3:** Correlation coefficients of the parameters of clot formation with laboratory tests of systemic inflammation in SLE patients by or near the time of examination.

	**Fibrinogen**	**Erythrocyte sedimentation rate**	**Albumin**	**α-2 globulin**	**β-1 globulin**	**β-2 globulin**	**Circulating immune complexes**
Lag	0.4	0.3	−0.3	−0.1	0.2	0.3	−0.0
V	0.6^*^	0.7^*^	−0.5	0.2	0.4	0.0	0.5
A (max)	0.7^**^	0.7^**^	−0.7^**^	0.7^*^	0.6^*^	0.9^*^	0.7^*^

* *p < 0.05*;

** *p < 0.01*.

**Table 4:** Correlation coefficients of the viscoelastic characteristics of plasma clots with the parameters of clots dynamic turbidimetry and laboratory tests of systemic inflammation in SLE patients by or near the time of examination.

**Parameters of visco-elasticity**	**Laboratory signs of systemic inflammation**					**Parameters of clot formation measured by turbidimetry**	
	**Fibrinogen**	**Erythrocyte sedimentation rate**	**β-2 globulin**	**Abs to β-2 glycoprotein**	**IgM**	**Polymerization rate**	**Maximum absorbance (A_**max**_)**
*G'*	0.6^**^	0.6^*^	0.7^*^	0.7^*^	0.6^*^	0.8^***^	0.9^***^
*G”*	0.5^*^	0.6^*^	0.6	0.7	0.6^*^	0.9^***^	0.8^***^
*G”/G'*	−0.6^**^	−0.3	−0.6	−0.3	0.03	−0.6	−0.5

* *p < 0.05*;

** *p < 0.01*;

*** *p < 0.001*.

### Fibrin Ultrastructure in Plasma Clots From SLE Patients

**Figure 5:**
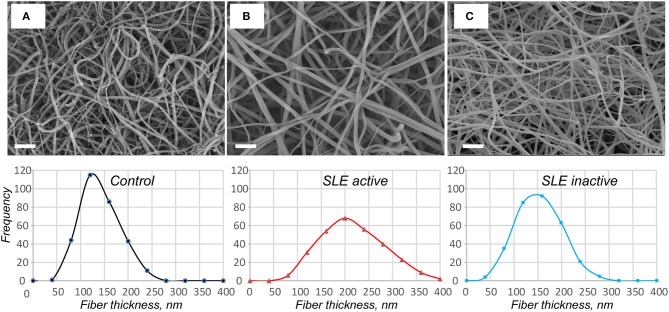
Representative scanning electron micrographs of fibrin clots formed from the control pooled normal plasma **(A)**, active **(B)**, and inactive **(C)** SLE plasma samples upon re-calcification. The fibrin structure in control and inactive SLE clots is more compact with smaller intrinsic pores. The fibers in active SLE clots are thicker with a reduced number of branch points. Magnification bars = 1 μm. In the lower row the corresponding distributions of fiber thicknesses are presented based on 300 individual fiber measurements (100 × 3 clots) per each experimental condition.

Using scanning electron microscopy, we studied the structural basis for the distinctions revealed in the absorption coefficient between the plasma clots in the active and inactive SLE groups and controls (Figure 5). Scanning electron microscopy showed significant differences in fibrin clot structure associated with SLE activity. The clots from the plasma of patients with the active form of SLE were much more porous, had fewer branch points and were built of significantly thicker fibers with an average diameter of 204 ± 44 nm compared to the clots formed in control plasma that were relatively compact and had thinner fibers with an average diameter of 129 ± 38 nm (*p* < 0.05) (Figures 5A,B). Clots from the plasma of inactive SLE patients comprised a dense network of thin fibrin fibers (138 ± 27 nm) without a significant difference from the control clots, but significantly thinner than clots from active SLE patients (*p* < 0.05) (Figure 5C). Unlike in control clots and clots from the plasma of inactive SLE patients, in clots from the plasma of patients with the active form of SLE, the thickness of fibrin fibers had a much broader distribution (Figure 5, lower panel), reflecting variability in thrombin generation and other conditions of clot formation in SLE.

### Two Types of Viscoelastic Behavior of Plasma Clots From SLE Patients

Shear rheometry of clots formed in plasma of SLE patients revealed two types of mechanical behavior. One (named type I, *n* = 14) was characterized by a significant increase of clot stiffness reflected by the storage modulus (*G*′ = 384 ± 69 Pa vs. *G*′ = 262 ± 9 Pa in control, *p* < 0.001) and a parallel increase of clot viscosity reflected by the loss modulus (*G*″ = 14.8 ± 2.3 Pa vs. *G*″ = 10.1 ± 0.5 Pa in control, *p* < 0.001). At the same time, the gels showed no significant changes in the relative viscosity/elasticity calculated as loss tangent (*tan* δ = 0.033 ± 0.004 vs. tan δ = 0.038 ± 0.020 in control, *p* > 0.05). Because clot viscoelasticity depends strongly on the amount of fibrin, we corrected the *G*′ and *G*″ values for fibrinogen concentration in the plasma samples with respect to the logarithmic dependence of *G*′ and *G*″ on fibrinogen concentration (39). After correction for fibrinogen levels by calculating *G*′/log[Fg] and *G*″/log[Fg], the difference in *G*′ and *G*″ between SLE and control clots became statistically insignificant (*G*′ = 735 ± 220 Pa for SLE vs. *G*′ = 629 ± 24 Pa for control and *G*″ = 28.3 ± 8.8 for SLE vs. *G*″ = 24.0 ± 0.7 for control, *p* > 0.05 for both). The slight difference in loss tangent also remained insignificant. These results showed that the observed distinction between storage and loss moduli in the SLE clots of type I and control clots were solely due to the higher fibrinogen concentration in the blood of SLE patients.

Type II mechanical behavior of SLE plasma clots (*n* = 14) was characterized by a significant decrease in both storage modulus (*G*′ = 157 ± 36 Pa in SLE vs. *G*′ = 262 ± 9 Pa in control, *p* < 0.001) and loss modulus (*G*″ = 7.1 ± 1.5 in SLE vs. *G*″ = 10.1 ± 0.5 Pa in control, *p* < 0.001), but a relatively greater ratio of the viscosity to elasticity as revealed by a higher value of loss tangent (*tan* δ = 0.045 ± 0.013 in SLE vs. *tan* δ = 0.038 ± 0.020 in control, *p* < 0.05). After correction for fibrinogen concentrations, the significant difference remained for *G*′ (*G*′ = 437 ± 164 Pa in SLE vs. *G*′ = 629 ± 24 Pa in control, *p* < 0.01), but turned out to be insignificant for *G*″ (*G*″ = 20.0 ± 7.7 Pa for SLE vs. *G*″ = 24.0 ± 0.7 Pa in control, *p* > 0.05). The loss tangent was elevated in SLE at the border line of significance (*p* = 0.05). These data show that the smaller stiffness of the type II clots, unlike clot viscosity, was not solely related to the lower fibrinogen concentration. The type I and type II clots had significantly different storage (*p* < 0.001) and loss moduli (*p* < 0.001) as well as the loss tangent (*p* < 0.05) both before and after correction for fibrinogen concentrations in the plasma samples analyzed.

**Figure 6:**
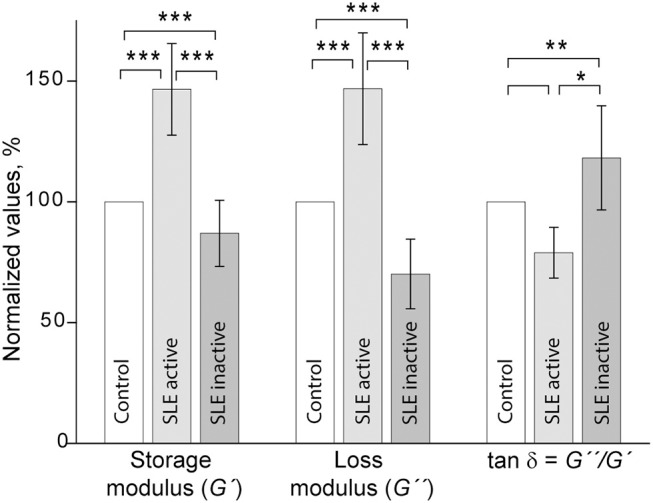
Comparative parameters of shear rheometry of plasma clots from patients with active and inactive SLE measured in parallel with control. The results are presented as the normalized mean value ± SD from the measurements of clots from control normal pooled plasma (taken as 100%) in parallel with clots from the SLE patients in the active (*n* = 14) and inactive (*n* = 14) forms of the disease. ^*^p < 0.05, ^**^p < 0.01, ^***^p < 0.001.

When the two types of clot mechanical behavior were analyzed with respect to clinical manifestations of the disease, it turned out that all of the type I clots with higher stiffness were observed in the plasma of patients with the active form of SLE, while all of the weaker type II clots were found in plasma of patients with the inactive form of SLE (Figure 6). Based on the structural data showing a more porous network with thicker fibers in the active form of SLE (Figure 5), this is apparently backwards from what would be expected since clots with thicker fibers and fewer branch points usually have lower *G*′ provided they have the same fibrin(ogen) concentration (25). However, fibrin viscoelasticity (as well as susceptibility to enzymatic lysis) can be determined by conditions other than fibrin structure, such as the protein mass fraction of a clot, the extent of crosslinking, and the presence of pathological plasma components, such as immunoglobulins and cellular microvesicles (40–42). Remarkably, correlation analysis of the viscoelastic characteristics of plasma clots with laboratory signs of immune inflammation confirmed that the mechanical properties (especially the viscosity) of plasma clots strongly and significantly correlated with the signs of acute phase of SLE assessed by erythrocyte sedimentation rate, the levels of fibrinogen, β-2 globulin, antibodies to β-glycoprotein 1, and IgM (Table 4). The viscoelastic parameters of plasma clots displayed also a strong association with the rate of fibrin polymerization and the optical density of clots, reflecting the fibrin network formation and structure (Table 4).

### Kinetics of the γ-γ-Crosslinking of Fibrin in Plasma Clots From SLE Patients

**Figure 7:**
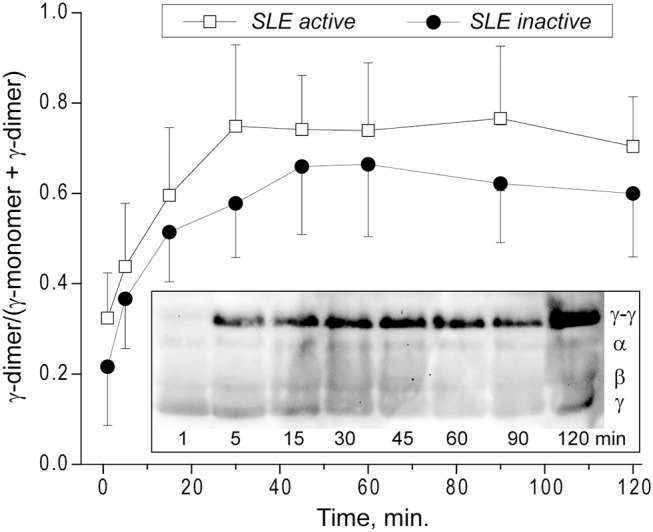
Kinetics of factor XIIIa-catalyzed γ-γ-crosslinking of fibrin in the plasma from SLE patients with active (*n* = 5) and inactive (*n* = 8) forms of the disease represented as a fraction of γ-dimer of the γ-chain-containing bands. The data at each point are shown as a mean ± SD. ***Insert:***Representative SDS-PAGE of the SLE plasma samples after addition of thrombin and CaCl_2_ that initiates both fibrin polymerization and factor XIII activation. The reactions were allowed to proceed for the desired time points followed by quenching with concentrated sample buffer containing a reducing agent and boiling. The products of the reaction were then run on an SDS-PAGE gel followed by Western blot analysis with an anti-fibrinogen polyclonal antibody. Lanes correspond to the time points of incubation.

Factor XIIIa-catalyzed covalent fibrin cross-linking of the γ and α chains has a dramatic effect both on the susceptibility to fibrinolysis and viscoelastic properties of blood clots (21). To see if the rate of factor XIIIa-induced crosslinking in fibrin clots is changed in the plasma of SLE patients, we measured the extent of γ-dimer formation as a function of time after thrombin-induced activation of clotting and factor XIII in the presence of Ca^2+^. The exogenous thrombin was added to make activation of FXIII and initiation of fibrin crosslinking independent of the rate of endogenous thrombin generation. The content of newly formed γ-dimers was determined using Western blot analysis of freshly formed reduced plasma clots and normalized by the sum of the intensity and width of the γ chain and γ-dimer bands at each time point (Figure 7). The rate and extent of γ-γ-dimerization shown as the averaged kinetic crosslinking curves was different between clots from the active and inactive SLE patients. The formation of γ-dimers went faster in the active SLE samples and reached a plateau at about 25 min, while in the inactive SLE samples the crosslinking was delayed and reached a plateau at about 45 min. The extent of γ-γ-crosslinking was consistently higher by 10–25% throughout the time of observation (120 min). Thus, the factor XIIIa-catalyzed γ-γ-dimerization of fibrin was enhanced in the clots obtained from the active SLE patients compared to those with inactive SLE, suggesting that the overall covalent fibrin stabilization, including γ- and α-chain crosslinking, may contribute the differences in the viscoelastic properties of fibrin that correlate with the severity of SLE.

## Discussion

Despite the fact that SLE patients are at increased risk of both arterial and venous thrombosis (6), the mechanisms of this predisposition as well as compensatory or protective antithrombotic reactions in SLE have not been fully elucidated. Chronic autoimmune inflammation implicates multiple cellular and molecular blood components that directly or indirectly affect the hemostatic system (43–45). One such component is fibrinogen, an acute phase reactant with pro-inflammatory properties that is upregulated and undergoes structural changes in response to inflammation-associated factors, such as oxidative stress, antibodies, etc (24, 46). Hyperfibrinogenemia is a hallmark of autoimmune disorders, including SLE (47). Fibrinogen conversion to fibrin provides the mechanical scaffold of blood clots and thrombi; therefore a possible contribution of the elevated fibrinogen level to the pathogenesis of thrombosis in SLE is formation of stiff and friable clots that are less deformable and prone to embolization (21, 48, 49). In accordance with this presumption, our results revealed that in patients with active SLE, hyperfibrinogenemia is associated with clots that have higher stiffness and plasticity, while clots from SLE patients in remission with normal fibrinogen levels were characterized by a weaker fibrin network (Figure 6). Fibrinogen levels have been shown to be directly associated with clot elasticity and viscosity with higher concentrations leading to an increase of clot rigidity (39, 50). Stiffer fibrin prevents an appropriate extent of clot deformability in response to blood shear and vascular wall tension; moreover, the higher stiffness of fibrin fibers sensed by platelets leads to increased platelet activation, adhesion and spreading (51). Thus, the hardening of the fibrin network associated with hyperfibrinogenemia revealed in the active SLE patients provides prothrombotic features of blood clots.

The results of our study summarized in Table 5 show that SLE clots have remarkable functional and structural features other than viscoelasticity that can contribute to the pathogenesis of thrombotic complications in SLE. One of the most important properties of plasma clots from the blood of SLE patients is increased susceptibility to t-PA-induced lysis (Figure 3). In the active SLE group of patients, this result is consistent with the porous network structure and thicker fibers. The number of fibers per volume determines resistance to the enzymatic lysis rather than an individual fiber diameter and less compact fibrin clots containing thicker fibers are more susceptible to lysis (52). Clots with fewer fibers have a higher t-PA/fiber ratio, resulting in increased fibrinolysis (48). This may account for the accelerated lysis in the active SLE clots, which have a decreased fiber density compared with healthy controls. However, clots from inactive SLE group, which had no structural differences from control clots, were also more lysable, although *in vitro* studies have reported that clots composed of thinner and denser fibers, are more resistant to fibrinolysis (48, 53–55). However, several studies have shown an opposite effect, so that clots made of thin fibers were cleaved faster than those made of thick fibers (3, 9, 56–58). These contrasting results likely come from the interplay between the movement of fibrinolytic agents (plasminogen, plasmin, t-PA, etc.) within a clot, the activity of those agents on fibers upon binding (40). Whether fine clots lyse faster or slower than coarse clots is determined by the number of t-PA molecules relative to the surface area of the clot exposed to those molecules (in conjunction with the number of fibers in the clot); therefore, at low t-PA concentrations coarse clots are lysed more rapidly but at high t-PA concentrations fine clots are lysed faster, which is another likely explanation for the conflicting data in the literature (59).

**Table 5:** Summary of the main plasma clot characteristics in SLE.

**Parameters studied**	**Active SLE**	**Inactive SLE**
	**(SLEDAI>4)**	**(SLEDAI<4)**
**Fibrinolysis**		
Total lysis time	↓	↓
Fibrinolysis rate	↑	↑
Clot lysis time	↓	↓
Fibrin(ogen) concentration [Fg]	↑	N
**Kinetics of Clot Formation**		
Lag time	↑	↑
Rate of fibrin polymerization	↑	↓
Maximal optical density (Amax)	↑	N
Maximal optical density (Amax) after correction for [Fg]	↑	N
**Clot Structure**		
Network density	↓	N
Branch points	↓	N
Fiber thickness	↑	N
**Clot Viscoelasticity**		
Storage modulus (*G′*)	↑	↓
Loss modulus (*G″*)	↑	↓
Loss tangent (*tan* δ = *G″/G′*)	N	↑
**Clot Viscoelasticity After Correction For [FG]**		
Storage modulus (*G′*)	N	↓
Loss modulus (*G″*)	N	N
Loss tangent (*tan* δ = *G″/G′*)	N	↑
Rate of fibrin crosslinking	↑	N

*↑ and ↓ show a significant increase or decrease, respectively, compared to clots from control normal plasma; N, no significant difference compared to clots from control normal plasma*.

The rate and degree of factor XIIIa-mediated fibrin crosslinking can also affect the lytic stability of fibrin. In addition to covalent attachment of α2-antiplasmin that has a strong antifibrinolytic potential, crosslinking of α and γ chains also reduce the fibrinolytic susceptibility of clots (60), although this is not always the case (61). In particular, fibrinolytic rates have been shown to be strongly regulated by γ chain crosslinking (62) that in our study was associated with higher SLE activity (Figure 7).

The general state of the fibrinolytic system may be another mechanism underlying the lytic stability of fibrin clots. Data on impairments of the fibrinolytic system in SLE are controversial (15). Some of them report increased levels of plasminogen (63), t-PA (17, 64), and hyperfibrinolysis (65), while others observed normal level of plasminogen and reduced level and activity of t-PA in SLE patients (66). It might be speculated that the greater susceptibility to lysis observed in the blood of SLE patients can comprise a protective and/or compensatory antithrombotic mechanism developed in response to suppression of the fibrinolytic potential and other pathogenic factors that predispose to thrombosis.

The changes in clot mechanics in active SLE patients were associated with altered clot structure (Figure 2) and enhanced covalent crosslinking (Figure 4) rendering fibrin stiffer (40, 67). Crosslinking of α chains is more important for stiffness than γ-dimerization, although the γ-dimerization is much faster, but there is ultimately a direct correlation between the extents of γ- and α-chain-crosslinking (21). In our study, the higher values of *G'* and *G”* were associated with more porous fibrin networks, fewer branch points, and thicker fibers, while usually such clots are less stiff (25). Although this is true as a generality, there at least four conceivable explanations for the increased fiber width and stiffness in the blood clots of active SLE patients. *First*, it is a result of a high fibrinogen level known to enhance fibrin fiber diameter (39); in contrast, in the patients with inactive SLE, thinner fibers correlate with lower fibrinogen concentrations (Table 3). The higher fibrinogen level may also cause an increase in the rate and degree of factor XIIIa-catalyzed covalent crosslinking (Figure 7) by providing more protofibrils nearby to enhance the formation of α-polymers further stabilized by α-chain crosslinking. *Second*, under acute phase conditions where the synthesis rate of fibrinogen is known to be enhanced, the degree of its phosphorylation considerably increases (68). The level of phosphorylated fibrinogen in SLE was found to be significantly higher than in healthy donors (69) and an increase in protein-bound phosphate markedly augments the thickness of the fibrin fibers (70). *Third*, thicker fibrin fibers are formed as a result of delayed thrombin generation (71) that was observed as a prolonged lag phase in the clotting turbidimetric curves that was more substantial in active than in inactive SLE (Figure 4). Fibrin polymerization at a lower thrombin concentration results in formation of thicker fibers due to slower elongation and greater lateral aggregation of protofobrils, while a higher level of thrombin results in a fine network of thin fibers (28, 72). The slower thrombin generation in SLE is likely due to the inhibitory effect of autoantibodies. A number of studies have shown interactions of aPL Abs with thrombin, factors IXa, Xa, and other serine proteases (73, 74) and IgG autoantibodies from SLE patients are shown to directly inhibit factor Xa (75). Misbalance in pro- and anticoagulants can also be an underlying mechanism, e.g., circulating plasma thrombomodulin was shown to be increased in SLE (76). *Fourth*, there may be a direct influence of plasma composition on clot structure and viscoelastic properties (77, 78), e.g., plasma proteins adhering to fibrin may add to fiber thickness and elasticity (22). The potential modulators of fibrin clot structure are α-2 globulin, β-1 globulin, β-2 globulin, circulating immune complexes, and albumin known to modulate fibrin structure (79, 80). Since there was no correlation of the lag time, obtained from the turbidimetry assay with the levels of the inflammation markers (Table 3), their influence on clot structure was independent of thrombin generation. At the same time, they all displayed a high correlation with the structure-related parameters, such as the maximal optical density of clots and clot mechanics (storage modulus) (Tables 3, 4).

The results of this study could be potentially influenced by including 3 active SLE patients with APS (Table 1), a prothrombotic condition that in known to affect by itself the formation, structure and properties of fibrin clots. However, excluding the APS patients from data analysis changed the results insignificantly, suggesting that APS can somewhat contribute but not overwhelm the overall effects of SLE on fibrin. Also, given the lack of acute prothrombotic conditions at the time of examination (Table 1), changes in fibrin clot properties revealed in this study are related to SLE *per se* rather than to other temporary pathological states or comorbidities. A limitation of this study that complicates pathophysiological interpretation of the results is that *in vivo* clots and thrombi, unlike clots formed *in vitro*, experience pro- and anticoagulant effects of vascular and blood cells. Furthermore, mechanical forces from the flow of blood especially in arteries, which tend to be more frequent sites of fatal thrombosis in SLE (81) affect significantly formation, structure and properties of fibrin networks *in vivo*.

In conclusion, we have demonstrated that lytic stability, formation, structure, and mechanical properties of fibrin clots are significantly altered in SLE and these changes generally correlate with the disease severity. In the blood of patients with a higher disease activity, hyperfibrinogenemia in combination with the delayed fibrin polymerization result in formation of a more porous fibrin network with thicker fibers that build clots that are lysed faster. In SLE patients with a lower disease activity, the fibrin network is less stiff and prone to irreversible plastic deformations while being still more lysable that control normal clots. The differences revealed in fibrin clot structure and properties between active and inactive SLE patients and normal subjects provide a pathogenic basis of predisposition to thrombosis in the active phase of SLE.

## Data Availability

All datasets generated for this study are included in the manuscript and/or the supplementary files.

## Ethics Statement

This study was carried out in accordance with the recommendations of the Ethical Committee of the Kazan State Medical University with written informed consent from all subjects. All subjects gave written informed consent in accordance with the Declaration of Helsinki. The protocol was approved by the Ethical Committee of the Kazan State Medical University.

## Author Contributions

JW and RL designed the research. RN, MS, and IA performed the experiments. RL, LZ, and JW analyzed data and wrote the paper. All authors reviewed and approved the final version of the manuscript.

### Conflict of Interest Statement

The authors declare that the research was conducted in the absence of any commercial or financial relationships that could be construed as a potential conflict of interest.
